# Tunnel Infection and Peritonitis Induced by *Staphylococcus aureus* Due to Decubitus Change of the Anterior Abdominal Wall in a Patient on Peritoneal Dialysis: Case Report

**DOI:** 10.3390/microorganisms12122608

**Published:** 2024-12-17

**Authors:** Marko Baralić, Ana Bontić, Jelena Pavlović, Vidna Karadžić-Ristanović, Selena Gajić, Jovan Jevtić, Pavle Popović, Kristina Petrović, Lara Hadži-Tanović, Aleksandra Kezić

**Affiliations:** 1 Clinic of Nephrology, University Clinical Center of Serbia, Pasterova 2, 11000 Belgrade, Serbia; 2 Faculty of Medicine, University of Belgrade, Dr Subotića Starijeg 8, 11000 Belgrade, Serbia; 3 Department of Pathology, Faculty of Medicine, University of Belgrade, Dr Subotića Starijeg 8, 11000 Belgrade, Serbia; 4 Clinic for Emergency Surgery, Emergency Center, University Clinical Center of Serbia, Pasterova 2, 11000 Belgrade, Serbia

**Keywords:** abdominal wall ulcer, peritoneal dialysis, *Staphylococcus aureus*, vancomycin

## Abstract

The occurrence of anterior abdominal wall ulcer at the site of the peritoneal catheter (PC) is one of the rarest complications of peritoneal dialysis (PD). When present, it is mainly caused by *staphylococci* which respond well to vancomycin therapy. Despite well-conducted therapy, there is a tendency to relapse and induce peritonitis, which makes it necessary to remove the PC and change the dialysis model of treatment and/or re-insert the catheter at another place to preserve PD as a treatment method. In the present study, we discuss a case of a 53-year-old patient with end-stage kidney disease treated with PD and with decubitus changes at the PC exit site; the change occurred due to migration of the catheter middle part by protruding from the abdominal cavity to the skin, thus allowing ulcer appearance. Although the PC site was treated with antibiotics, as advised by the surgeon, the patient was finally transferred to hemodialysis as the repositioning of the catheter was not performed. This leads to the conclusion that the antibiotic treatment and catheter repositioning are mandatory to preserve peritoneal dialysis as an end-stage kidney disease (ESKD) treatment model.

## 1. Introduction

Chronic kidney disease (CKD) is a global public health issue of increasing concern, responsible for 1.2–1.3 million deaths in 2017. The mortality rate from CKD has increased by 41.5% in the last three decades. All the above points to the development of end-stage kidney disease (ESKD) and the need for treatment including kidney replacement therapy (KRT) such as chronic peritoneal dialysis (PD) [1,2].

PD is a common treatment method for the elderly, who are more prone to infections, as well as patients with pronounced cardiovascular comorbidities, which is more than 50% of all dialysis patients. PD is one and the best model of ESKD treatment for younger, active patients, especially in countries with a large area and rare hemodialysis (HD) trim such as the USA, Canada, Australia, Hong Kong, and Mexico [1,2,3]. The most common mortality pattern in patients on PD is caused by recurrent infections of the peritoneal catheter (PC) exit site and peritonitis [4,5]. Recent research shows that compared to the general population, patients on PD are more prone to oxidative stress and chronic inflammation that in turn lead to an increased risk of cardiovascular disease [6], and possibly ischemia of the subcutaneous tissue due to an increase in intracapillary pressure [7]. In addition to the above, they are prone to anemia like other patients with ESKD, due to which hypoperfusion of the skin and subcutaneous tissue is possible, as well as secondary hyperparathyroidism, which can lead to calciphylaxis that impairs the integrity of the skin [8]. Ulcerations represent the most severe skin defect and a significant clinical problem associated with increased mortality. They are characterized by local inflammation and pain edema and are an excellent substrate for colonization by saprophytes. The healing of such changes is usually long and requires intensive toileting [9,10,11].

In the present case study, we wanted to demonstrate our experience in treating a 53-year-old male with a diagnosis of ESKD who is on PD treatment and with recurrent decubitus changes caused by PC. The patient developed abdominal wall ulcer, which is the rarest mechanical complication, especially considering that the change occurred due to the migration of the catheter middle part by protruding from the abdominal cavity to the skin, thus allowing ulcer appearance.

## 2. Case Presentation Section

The case study was approved by the Ethical Committee of the University Clinical Centre of Serbia (approval number: 890/8) and conducted following the Declaration of Helsinki and the Ethical Guidelines for Medical and Health Research Involving Human Subjects. Patient informed consent was obtained.

**Figure 1:**
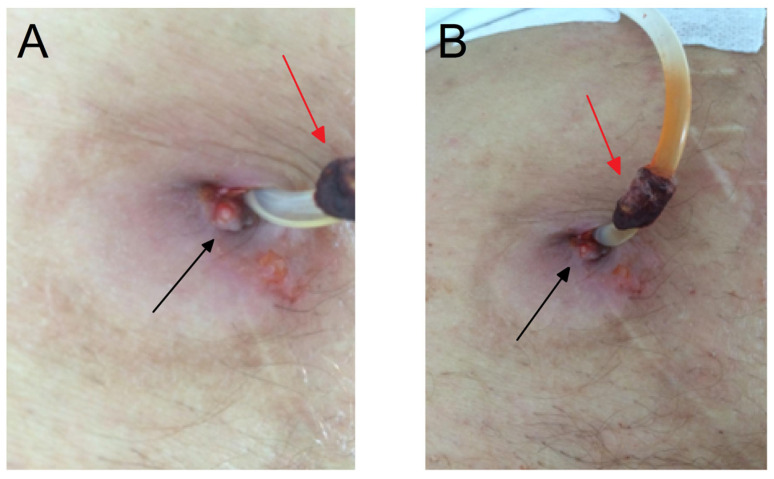
An extruded subcutaneous cuff (red arrow) ulceration and granulation tissue (black arrow) present on peritoneal catheter exit site. A photo was taken from two angles to clearly represent the observed change (**A**,**B**).

This is a 53-year-old patient whose underlying disease is a right testicular tumor that was discovered at the age of thirty. After one-year post orchiectomy, metastatic changes in the lungs occurred, and sternotomy was carried out with complete extirpation of the tumor change. The patient was treated with chemotherapy but, after the fourth cycle (out of six planned), azotemia occurred with creatinine values of 900 µmol/L, resulting in chemotherapy discontinuation after which renal function recovered incompletely with creatinine values of 120 µmol/L (CKD grade 3). Besides suffering from a chronic kidney disease for about 20 years, the patient was diagnosed with hypertension (HTN) 10 years ago and was treated for congestive heart failure. In addition, he began treatment with continuous ambulatory PD with 1.36% glucose solutions 4 times a day. The first episode of abdominal wall ulcer in the immediate vicinity of the PC appeared in November in 2016, with a 2.0 cm diameter of ulcerative change, showing skin with redness (Figure 1).

Except for the change in the abdominal skin, other physical findings were not detected. Biochemical analyses at the first hospitalization are presented in Table 1. During the hospitalization, both the abdominal and plastic surgeons agreed that the change should only be treated locally, with no indication for surgical treatment being necessary. The peritoneal dialysate sediment was clear without the presence of leukocytes. The swab of the PC exit site was sterile as well as the peritoneal dialysate culture. The second hospitalization, which followed after 2 months, resulted in the visible prominence of the PC at a distance of 4.0 cm from the tunnel of the PC exit site. At this point, the swab culture was invocative of *Staphylococcus aureus*, which was shown to be sensitive to vancomycin. Nevertheless, after 2 weeks, the first episode of peritonitis occurred, and the patient was treated with vancomycin for three full weeks. The dialysate culture was again positive to *S. aureus*. Standard protocol at the University Clinical Center of Serbia is the introduction of systemic antimicrobial therapy once the concentration of bacteria is >100,000 CFU/mL and the antibiotic of choice is vancomycin, due to its potent effect on all *S. aureus* strains. The therapy lasts 2–3 weeks and its success is monitored through the analysis of dialysis sediment. Every two days, dialysis sediment is checked for the presence of leukocytes; if their number is >30, the therapy is continued until the leukocyte number drops below 5, indicating successful treatment and the curation of peritonitis as well as the end of therapy.

**Figure 2:**
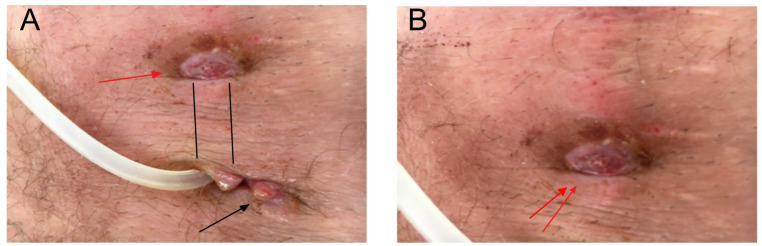
The granulation tissue (black arrow) at the catheter exit site and ulceration (red arrow) (**A**) in the catheter tunnel region (the path of the catheter tunnel is marked with two parallel lines) (**B**).

**Figure 3:**
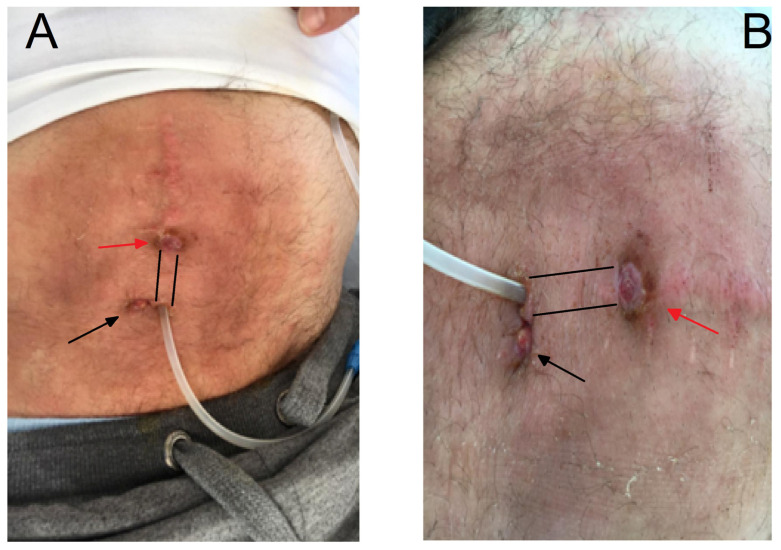
The granulation tissue (black arrow) at the catheter exit site and ulceration (red arrow) (**A**) in the catheter tunnel region (the path of the catheter tunnel is marked by lines) (**B**).

Biochemical analyses at the second hospitalization are also given in Table 1. After the remedial infection, a PC was removed and the patient was transferred to another modality of ESKD treatment, i.e., HD. The PD sediment was abundant in leukocytes, whereas the dialysate culture as well as the PD exit site swab were rich in *S. aureus* (Figure 2 and Figure 3).

## 3. Discussion

Decubitus skin alteration of the anterior abdominal wall due to elevated pressure from the PC tunnel is a rare complication of PD. Delayed abdominal wall ulcer perforation of a viscus is explained by the intimate contact between the PC and the viscus, due to the close contact between the PC and the tunnel, causing continuous pressure that can end in a decubitus erosion or perforation. It is assumed that constant pressure leads to ischemia of the subcutaneous tissue. The mean pressure in the skin capillaries is 25 mmHg. When the pressure is greater than 30 mmHg, compression changes occur, causing ischemia that leads to tissue hypoxemia and necrosis, while the tissue necrosis causes the abdominal wall ulcer to develop. Additional deterioration can occur due to severe anemia in this group of patients. Tunneled change is also a good basis for the development of infection due to the existence of the outer part of the PC [12]. It especially refers to saprophytic microorganisms and previous tunnel infections in shorter periods of time, as well as the more frequent use of antimicrobial creams [13,14,15]. In addition to tunnel infections, risk factors include physical inactivity, age, immobilization, malnutrition, obesity, wearing wide belts, poor hygiene, use of diapers, etc. [16,17,18].

*S. aureus*, a regular inhabitant of the bacterial flora of skin and mucous membranes, rarely causes infections on healthy skin, but if bacteria find its way into the bloodstream or internal tissues, it may lead to serious infections [19]. Treatment depends on the bacterial strain as well as the type of infection. The constant and, above all, inadequate usage of antibiotics leads to the formation of resistant bacterial strains. Penicillin is the drug of choice, but in the methicillin-resistant *S. aureus* strain (also known as MRSA), the treatment is carried out with vancomycin [20]. Antimicrobial tolerance can be attributed to the presence of coinfecting microorganisms, i.e., *Candida albicans* as well as quorum-sensing molecule farnesol, secreted by *C. albicans*. *C. albicans–S. aureus* mixed biofilm formation on medical devices leads to increased infections [21]. Depending on the results of the antibiogram and the sensitivity of isolated bacteria, other antibiotics can be employed as well. Besides antibiotics, daily care and disinfection of the ulcerous change as well as other skin wounds are desired. In addition to antimicrobial therapy and depending on the type of infection, other procedures are often included, such as the removal of prosthetic devices (e.g., catheter) or fluid replacement management. In patients on PD, frequent control of the PC exit site swabs is necessary, even when there is no external inflammation, due to possible tunnel infections, especially in the sensitive population, which includes patients with malignancy or malnutrition. In patients with normal serum albumin status and global hypoproteinemia, it is necessary to advise a protein-rich diet, especially if they have a lower body mass index as it is an indicator of malnutrition, which can accelerate the development of decubitus ulcers, as well as the best possible correction of anemia and volume status in order to prevent subcutaneous tissue edema and weaker epithelialization [22]. The patient analyzed in this study had almost all aforementioned risk factors for lesion formation.

Most skin wall ulcers are caused by the venous system, but they can occur as part of systemic inflammation or chronic malnutrition. Chronic skin wall ulcers are often called “inflammatory ulcers” such as pyoderma gangrenosum, leukocytoclastic vasculitis, and cryoglobulinemic vasculitis [23]. The presented patient had no changes corresponding to those described, and although the catheter cuff was dislodged, this rare complication of abdominal wall ulcer was formed by penetrating through the subcutaneous and skin tissue in the area of the catheter distant from the cuff.

## 4. Conclusions

Pressure sores and local exit site infection may not require catheter replacement if they respond to antibiotics and wound care. Stronger indications of removal or replacement of the catheter are peritonitis and extrusion of the catheter cuff. Therefore, all decubitus changes need to be treated surgically, because even if the local infection is successfully fought, further complications in the form of peritonitis are likely to occur. In the presented case, required steps were taken, i.e., treatment of the PC site with antibiotics, as advised by the surgeon. Finally, the patient was transferred to hemodialysis as we did not conduct the repositioning of the catheter, leading to the conclusion that the antibiotic treatment and catheter repositioning are mandatory to preserve peritoneal dialysis as an ESKD treatment model.

## Figures and Tables

**Table 1:** Biochemical and hematological characterization.

*Concentration*	*First Hospitalization*	*Second Hospitalization*	*Reference Range*
**C-reactive protein** (**mg/L**)	71.8	176.2	1–8
**Leukocytes** (**×10^9^/L**)	6.4	12.7	3.6–10.0
**Erythrocytes** (**×10^12^/L**)	3.38	3.35	4.34–5.72
**Hemoglobin** (**g/L**)	98	101	138–175
**Hematocrit** (**L/L**)	0.31	0.32	0.41–0.53
**Total protein** (**g/L**)	58	63	62–81
**Albumin** (**g/L**)	42	40	35–53
**Glucose** (**mmol/L**)	5.5	4.8	4.4–6.2
**Urea** (**mmol/L**)	22.0	24.2	1–8
**Creatinine** (**μmol/L**)	486	1014	40–92
**Transferrin** (**g/L**)	1.7	/	1.7–3.8
**Ferritin** (**μg/L**)	227.0	264.0	30–400
**Iron** (**μmol/L**)	11.5	11.8	11–30
**Transferrin saturation** (**%**)	28.0	26.0	20–50

## Data Availability

The original contributions presented in the study are included in the article, further inquiries can be directed to the corresponding author.
